# Evaluation of Subclinical Left Ventricular Dysfunction in HIV Patients Receiving Abacavir, Dolutegravir, and Lamivudine Therapy with Novel Tissue Doppler Imaging Techniques

**DOI:** 10.3390/jcm14051534

**Published:** 2025-02-25

**Authors:** Dogac Oksen, Muzaffer Aslan, Ebru Serin, Muhammed Heja Gecit, Yunus Emre Yavuz, Esra Yerlikaya Zerdali, Veysel Oktay

**Affiliations:** 1 Department of Cardiology, Medical Faculty, Altinbas University, Istanbul 34217, Turkey; 2 Department of Cardiology, Medical Faculty, Siirt University, Siirt 56100, Turkey; mzfraslan56@hotmail.com (M.A.); yemre91@icloud.com (Y.E.Y.); 3 Department of Cardiology, Sisli Hamidiye Etfal Training and Research Hospital, Istanbul 34418, Turkey; serin.ebru@hotmail.com; 4 Department of Cardiology, Cerrahpasa Institute of Cardiology, Istanbul University, Istanbul 34467, Turkey; gecitheja@gmail.com (M.H.G.); drvoktay@gmail.com (V.O.); 5 Department of Infectious Diseases and Clinical Microbiology, Istanbul Haseki Research and Training Hospital, Istanbul 34096, Turkey; esrayerlikaya@gmail.com

**Keywords:** highly active antiretroviral therapy, human immunodeficiency virus, myocardial performance index, isovolumic acceleration, tissue Doppler imaging, subclinical ventricular dysfunction

## Abstract

**Background/Objectives:** Highly active antiretroviral therapy (HAART) effectively suppresses viral load and aids immunological recovery in HIV patients, but may still lead to subclinical myocardial dysfunction. This study assesses left and right ventricular functions in patients on HAART containing abacavir, dolutegravir, and lamivudine using Tissue Doppler Imaging (TDI). **Methods**: This observational cross-sectional study involved 118 HIV-positive adults on HAART and 80 age- and gender-matched healthy controls. Comprehensive echocardiographic assessments, including TDI, were conducted to evaluate myocardial performance index (MPI) and isovolumic acceleration (IVA). **Results**: Conventional echocardiographic parameters showed no significant differences; however, TDI indicated significant impairments in ventricular functions in the HAART group, with increased MPI and decreased IVA (*p* < 0.001). Pulmonary artery pressures were also higher in the HIV group (*p* = 0.012). There was a strong positive correlation between MPI and HAART duration (r = 0.675, *p* = 0.002), and a negative correlation with CD4 count (r = −0.545, *p* = 0.006). **Conclusions**: TDI reveals significant subclinical ventricular dysfunction in HIV patients on HAART, correlating with therapy duration and immune status. These findings underscore the utility of TDI in detecting myocardial deterioration before clinical symptoms appear.

## 1. Introduction

In our increasingly globalized world, where human life expectancy continues to rise, the prevalence of human immunodeficiency virus (HIV) is also increasing. As access to effective treatments becomes easier, the number of people living with HIV (PLWH) who can lead normal lives in society despite having the disease also increases [1]. HIV can lead to myocardial damage either directly, via myocardial invasion, or indirectly, via opportunistic infections [2]. With the introduction of highly active antiretroviral therapy (HAART), the prevalence of cardiomyopathies, which had previously reached up to 30%, has decreased significantly [3]. Despite the significant reduction in cardiovascular manifestations achieved with HAART, cardiomyopathies remain a worrisome complication in PLWH, leading to long-term adverse outcomes and prolonging patient follow-up. Combination therapy containing abacavir, dolutegravir, and lamivudine among HAART regimens has proven effective in suppressing viral load and improving immunological recovery, as shown in the Study ING114467 (SINGLE), where it reduced HIV-1 ribonucleic acid (RNA) levels to less than 50 copies per milliliter [4]. Nonetheless, studies have shown that abacavir, a component of this combination therapy, increases the risk of cardiovascular diseases, particularly myocardial infarction [5]. Additionally, several studies have shown an increase in intima–media thickness and the risk of premature atherosclerosis in patients receiving HAART [6,7]. Therefore, despite the high efficacy of the combination therapy containing abacavir, dolutegravir, and lamivudine in the commonly used HAART regimens, its long-term effects, especially on cardiovascular health, remain a matter of concern and active research. The fact that the left ventricular ejection fraction (LVEF), which is commonly used in the evaluation of left ventricular dysfunction, may remain unchanged until the late stages of the disease may delay the detection of subclinical dysfunction [8]. Tissue Doppler Imaging (TDI), an advanced echocardiography (ECHO) modality that allows for the precise assessment of myocardial tissue velocities, providing valuable insights into both systolic and diastolic ventricular functions, is an alternative technique that can detect subclinical dysfunction in earlier stages of the disease, even in the absence of overt cardiovascular symptoms. Myocardial performance index (MPI), a TDI-derived parameter combining systolic and diastolic time intervals, serves as an integrative measure of global ventricular function [9,10].

In view of the foregoing, the objective of this study is to assess subtle ventricular dysfunction in the left and right ventricular functions in PLWH without known cardiovascular pathology receiving combination therapy containing abacavir, dolutegravir, and lamivudine from HAART regimens using novel TDI techniques, with a view to enhancing cardiovascular monitoring and intervention strategies in this patient population.

## 2. Materials and Methods

### 2.1. Study Design

This study was designed as an observational cross-sectional single-center study. The study protocol was approved by the Local Ethics Committee of Istanbul University Cerrahpasa (no: B 08.06 50.0.05.00/14, date: 12 December 2018). The study was conducted in accordance with the ethical principles outlined in the Declaration of Helsinki and the Good Clinical Practice Guidelines. Written informed consent was obtained from the participants included in the study.

### 2.2. Population and Sample

The study population consisted of individuals over the age of 18 who were infected with HIV, compliant with HAART treatment, regularly followed up by the infectious diseases department, and referred to our clinic for cardiological evaluation between January 2019 and December 2023. Participants with congenital heart disease, known cardiovascular disease before treatment, substance abuse, poor compliance with follow-up and HAART treatment, and those who refused to participate in the study were excluded from the study. In the end, the patient group consisted of 118 PLWH on HAART, and the control group consisted of 80 healthy individuals matched for age and gender with the patient group.

### 2.3. Data Collection and Echocardiographic Assessment

Participants’ demographic characteristics, physical examination, and vital findings were recorded. Detailed ECHO examinations were performed by two expert echocardiographers using a Philips Affiniti 50 ECHO device with an S5 transducer in the two-dimensional, M-mode, color Doppler, and pulsed-wave (PW) Doppler modes while the patients were in left lateral and supine positions to obtain appropriate images. All ECHO recordings were conducted using subxiphoid imaging methods alongside single-lead electrocardiographic monitoring from the parasternal long-axis, short-axis, and apical two-, four-, and five-chamber views. LVEF was calculated using Simpson’s method. The dimensions of the left atrium (LA), right atrium (RA), and left ventricle (LV) during end-diastole and end-systole were assessed from the parasternal long-axis view, whereas the basal dimension of the right ventricle (RV) was assessed from the apical four-chamber view. E and A mitral inflow velocities and the E/A ratio were assessed using PW Doppler imaging from the apical four-chamber view. The Doppler cursor was positioned at the tips of the mitral valve leaflets during diastole. Tricuspid annular plane systolic excursion (TAPSE), used to evaluate RV systolic function, was measured with M-mode ECHO at the lateral tricuspid annulus during systole.

The peak myocardial velocity during isovolumic contraction (IVV), the acceleration time (AT) of the IVV, the peak myocardial systolic velocity (Sa), and the peak early (E′) and late diastolic velocities (A′) were measured. Isovolumic contraction time (IVCT), isovolumic relaxation time (IVRT), and ejection time (ET) were also recorded. MPI was calculated by dividing the sum of the IVCT and IVRT by ET. Isovolumic acceleration (IVA) was determined by dividing the isovolumic velocity (IVV) by AT, specifically using IVV and AT [11]. All ECHO images were digitally stored and analyzed offline per the established standards to ensure accuracy and reproducibility. All measurements were performed in accordance with the guidelines established by the American Society of Echocardiography and the European Association of Cardiovascular Imaging [12]. To minimize variability, the mean values of the measurements for each parameter during three consecutive cardiac cycles were calculated and recorded. Additionally, repeat measurements were performed on a subset of images to assess intra-observer variability in ECHO parameters. Sample sizes were calculated to detect a clinically significant difference in MPI between the HAART group and controls, anticipating a difference of 0.1 units with a standard deviation of 0.10. With an alpha of 0.05 and a power of 80%, a minimum of 63 subjects per group was required. To accommodate potential dropouts, we enrolled 118 patients and 80 controls [13].

### 2.4. Statistical Analysis

Statistical analyses of the collected data were conducted using the SPSS 27.0 (Statistical Product and Service Solutions for Windows, Version 27.0, IBM Corp., Armonk, NY, USA, 2020) software package. The results of the statistical analyses were expressed using descriptive statistics, i.e., means ± standard deviation values in the case of continuous variables determined to conform to the normal distribution, medians with interquartile ranges (IQR) in the case of continuous variables determined to not conform to the normal distribution, and frequencies (*n*) and percentage (%) values in the case of categorical variables. The normal distribution characteristics of the numerical variables were analyzed using the Shapiro–Wilk test. For comparing the differences in numerical variables between the two independent groups, the independent samples t-test was used for numerical variables determined to conform to the normal distribution and the Mann–Whitney U test was used for numerical variables determined to not conform to the normal distribution. Additionally, in comparing the differences in categorical variables between the groups, Pearson’s correlation coefficients were used to evaluate relationships between normally distributed continuous variables and Spearman’s rank correlation coefficients were used to evaluate relationships between non-parametric variables. All statistical tests were two-tailed. Probability (*p*) statistics of ≤0.05 were deemed to indicate statistical significance.

## 3. Results

### 3.1. Demographic and Clinical Characteristics

Both the patient and control groups consisted predominantly of males, and there was no significant difference between them in terms of gender (*p* = 0.280). There were also no significant differences between the groups in age, vital signs, or patient characteristics. In terms of comorbidities, hyperlipidemia was more common, albeit not significantly, in the patient group than in the control group (27.9% vs. 22.5%, *p* = 0.064) (Table 1).

### 3.2. Left Ventricular Echocardiographic Findings

A comparison of conventional ECHO parameters between the two groups revealed no significant differences in LV dimensions, wall thicknesses, and LVEFs (Table 2). On the other hand, diastolic function analysis revealed that the E mitral velocity was significantly lower in the patient group than in the control group (7.23 ± 2.87 cm/s vs. 8.68 ± 3.01 cm/s, *p* = 0.048), there was no difference between the groups in A mitral velocity, and the E/A ratio was significantly higher in the patient group than in the control group (0.86 ± 0.42 vs. 0.77 ± 0.40, *p* = 0.030).

The MPI of the LV was significantly elevated in the patient group compared to the control group (0.54 ± 0.08 vs. 0.43 ± 0.10, *p* = 0.001), suggesting subclinical global ventricular dysfunction. Additionally, AT was significantly higher and IVA was significantly lower in the patient group than in the control group (30.41 ± 5.10 ms vs. 26.97 ± 4.30 ms, *p* = 0.030; 3.70 ± 1.60 m/s^2^ vs. 4.30 ± 1.80 m/s^2^, *p* = 0.004).

### 3.3. Right Ventricular Echocardiographic Findings

RV analysis revealed significantly elevated mean pulmonary artery pressure (meanPAB) in the patient group compared to the control group (18.51 ± 6.00 mmHg vs. 13.69 ± 5.40 mmHg, *p* = 0.012). There was no significant difference between the groups in terms of TAPSE, a measure of RV systolic function (27.73 ± 3.50 mm vs. 27.11 ± 3.10 mm, *p* = 0.742). However, the MPI of the RV was significantly higher in the patient group than in the control group (0.64 ± 0.09 vs. 0.47 ± 0.08, *p* = 0.010), suggesting subclinical RV dysfunction. Additionally, the AT of the tricuspid annulus was significantly higher and IVA was significantly lower in the patient group than in the control group (35.40 ± 7.00 ms vs. 29.35 ± 6.50 ms, *p* < 0.001; 1.83 ± 0.95 m/s^2^ vs. 2.49 ± 1.00 m/s^2^, *p* < 0.001) (Table 3).

### 3.4. Correlation Analysis

**Figure 1:**
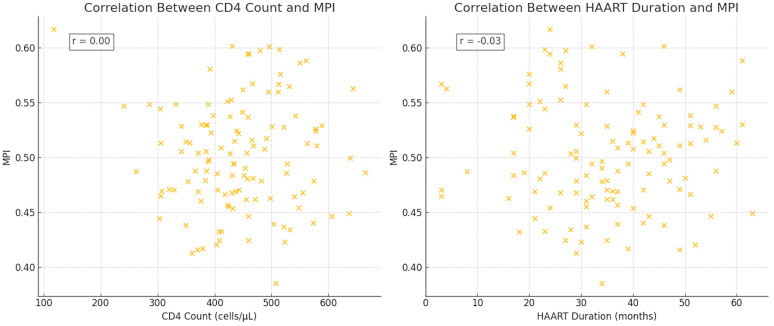
A scatter plot demonstrating the negative correlation between CD4 count and myocardial performance index (MPI) (r = −0.545, *p* = 0.006) and illustrating the positive correlation between HAART duration and MPI (r = 0.675, *p* = 0.002) in people living with HIV receiving HAART.

Correlation analysis revealed a moderate negative correlation between MPI and CD4 count (r = −0.545, *p* = 0.006), indicating that lower CD4 counts were associated with poorer global ventricular function, and a strong positive correlation between MPI and HAART duration (r = 0.675, *p* = 0.002), suggesting that longer treatment duration may contribute to subclinical myocardial dysfunction (Figure 1).

## 4. Discussion

Increasingly sensitive ECHO techniques have been developed to evaluate myocardial tissue function. The ability to detect functional impairment in the tissue without overt myocardial damage, clinical symptoms, or wall motion abnormalities is particularly critical in terms of cardiomyopathy-related diseases and treatment regimens that carry the risk of cardiomyopathy.

In this context, we compared the TDI ECHO findings of PLWH without cardiovascular disease undergoing effective HIV treatment featuring a HAART regimen to those of healthy individuals. Although HAART provides effective viral suppression and immunological recovery, subclinical myocardial dysfunction remains a significant concern in PLWH. While no significant differences were observed between the groups in conventional ECHO findings, both the LV and RV TDI findings indicated that diastolic function and subclinical myocardial dysfunction worsened significantly in the patient group compared to the control group. The RV and LV MPIs were significantly higher and the RV and LV IVAs were significantly lower in the patient group than in the control group. The MPI showed significant positive and negative correlations with HAART duration and CD4 count, respectively.

The role of HAART in the development of cardiovascular disease remains unclear. Some studies have suggested that HAART may predispose PLWH to developing cardiac dysfunction by disrupting myocyte metabolism [14,15]. On the other hand, it has been argued that HAART reduces the frequency of cardiovascular events by decreasing immune activation and suppressing inflammation. Persistent inflammation, however, has been reported to cause endothelial damage, thereby increasing the risk of disease-related cardiovascular events [16].

There are only a few studies in the literature that have evaluated subclinical systolic and diastolic functions in patients who are adherent to HAART and have their viral load under control [8]. In one of these studies, Schuster et al. reported that PLWH without cardiovascular disease and receiving antiretroviral therapy exhibited subclinical myocardial dysfunction, including early signs of diastolic dysfunction and ventricular remodeling [8]. In another of these studies, which evaluated children and young adults who were asymptomatically infected with HIV early in life using strain echocardiography, Sims et al. detected impaired systolic strain in individuals with normal LV function and LVEFs, as also shown by conventional methods [17]. Reinsch et al. detected left ventricular (LV) dilatation in 10.1% of 803 treated PLWH, septal hypertrophy in 18%, a reduced LVEF in one-third, and diastolic dysfunction in 48%. These rates were higher than the average of the respective rates reported in the literature [18]. While the mean duration from HIV infection to the time the study was conducted was 7.5 years in the said study, the mean duration of treatment in our study was 35 months. The discrepancies between studies’ respective findings may be attributed to the differences between the studies in terms of time from disease onset to the time the study was conducted. In a study comparing 21 PLWH on HAART with 27 healthy control subjects, Onur et al. found that, in the PLWH group, LVEF was significantly lower and septal and lateral systolic strain were significantly reduced, as demonstrated by primary assessment featuring strain imaging [19]. In comparison, in our study, while conventional ECHO evaluations revealed higher, albeit non-significant, LVEF and LV wall thickness and dimensions in the PLWH group compared to the control group, TDI ECHO evaluations revealed significant deterioration in myocardial performance parameters, especially MPI and IVA.

The prolonged AT and reduced IVA we observed in our study suggest impaired myocardial contractile efficiency. These findings are consistent with studies that have associated chronic inflammation and endothelial dysfunction with adverse myocardial remodeling in PLWH [7]. The significant correlation between MPI and HAART duration underscores the potential cardiotoxic effects of prolonged antiretroviral therapy. Similar concerns have been raised regarding the cardiovascular risks associated with specific HAART components, particularly abacavir, which has been linked to an increased risk of myocardial infarction [5].

Sitbon et al. reported that the etiology of pulmonary hypertension in PLWH is multifactorial, including HIV-related inflammation, co-infections, and the adverse effects of antiretroviral therapy [20,21]. Additionally, despite effective HAART, residual immune activation has been shown to play a role in vascular remodeling and increased pulmonary artery pressures [22]. In comparison, we detected a significantly higher meanPAB in the PLWH group compared to the control group, which is in line with data in the literature indicating that pulmonary hypertension is a prevalent complication in HIV patients, potentially attributable to chronic inflammation, endothelial dysfunction, and immune activation associated with the disease.

The strong negative correlation we found between MPI and CD4 count suggests that immunological status plays a critical role in the survival of myocytes. Lower CD4 counts, indicative of immune suppression, may exacerbate systemic inflammation and oxidative stress, contributing to myocardial dysfunction [23,24]. Conversely, the positive correlation we found between MPI and HAART duration points to the need for further investigation into the long-term cardiovascular effects of antiretroviral therapy.

In light of recent advancements, the integration of speckle-tracking echocardiography (STE) and myocardial work indices has emerged as pivotal for assessing subclinical ventricular dysfunction. Myocardial work analysis, as described by Trimarchi et al., provides a comprehensive and load-independent measure of myocardial function that transcends the capabilities of traditional echocardiographic parameters such as global longitudinal strain [25]. The integration of such advanced diagnostics is crucial for enhancing the detection and management of myocardial impairment in HIV patients, aligning with the need for refined monitoring strategies highlighted throughout our findings. In addition to ECHO techniques, computed tomography that allows for the evaluation of coronary artery disease, coronary calcium assessment, and other imaging tests indicating vascular condition such as arterial stiffness, can provide valuable clinical data in the group of people living with HIV who are at high cardiovascular risk. The early diagnosis and prevention of cardiovascular diseases are significantly enhanced by the assessment of the myocardium with ECHO, together with additional modalities [26].

### Limitations of the Study

Apart from its strengths, such as featuring a control group that was well matched to the patient group and a comprehensive ECHO assessment, there were some limitations to our study. First, while there was a control group consisting of healthy individuals, the absence of a control group comprising HIV-infected individuals who did not receive treatment for ethical reasons did not make it possible to evaluate the effects of HIV and HAART separately. Secondly, the study’s cross-sectional design prevented the establishment of a cause–effect relationship. Thirdly, the study was based solely on ECHO, did not utilize advanced imaging methods such as magnetic resonance imaging, and did not examine biomarkers such as N-terminal pro-B-type natriuretic peptide (NT-proBNP), a measure of myocardial function. Misclassification bias could arise from solely using echocardiographic assessments. Additionally, the potential for type I and type II errors exists due to the limited sample size and the number of statistical tests performed.

## 5. Conclusions

Our findings revealed subclinical ventricular dysfunction in PLWH receiving HAART, highlighting the importance of using advanced ECHO techniques in its early diagnosis. Measurements of MPI and IVA, two of the TDI parameters, revealed significant impairment in both LV and RV functions in PLWH receiving HAART that was otherwise undetectable by conventional ECHO measurements.

The correlations between MPI, HAART duration, and CD4 count suggest a complex interplay between HAART duration, immunological status, and myocardial health, underscoring the need for comprehensive cardiovascular monitoring in PLWH, even in asymptomatic patients. Future studies featuring longitudinal designs and integrating advanced imaging techniques and biomarker analyses are warranted to corroborate the findings of our study and further elucidate the mechanisms underlying these findings to guide therapeutic strategies. In conclusion, our study emphasizes the importance of using an integrated approach to cardiovascular care in PLWH in order to optimize their long-term health outcomes and quality of life.

## Figures and Tables

**Table 1:** Baseline demographic and clinical characteristics of people living with HIV on HAART versus healthy controls.

Characteristics	PLWH (*n* = 118)	Controls (*n* = 80)	*p*-Values
Age (years)	44.10 ± 12.67	43.97 ± 7.20	0.852
Male sex, *n* (%)	78 (66.1)	45 (56.2)	0.280
BMI (kg/m^2^)	24.32 ± 3.10	25.44 ± 3.55	0.156
Vital Signs			
DBP (mmHg)	78.26 ± 3.90	80.12 ± 5.54	0.324
SBP (mmHg)	120.20 ± 8.47	124.12 ± 7.94	0.098
Heart rate (bpm)	82.15 ± 5.23	78.12 ± 8.58	0.142
Comorbidities, *n* (%)			
Current smoking	65 (55.0)	28 (35.0)	0.043 *
Diabetes mellitus	22 (18.6)	12 (15.0)	0.307
Hypertension	17 (14.4)	13 (16.2)	0.462
Hyperlipidemia	33 (27.9)	18 (22.5)	0.064
HIV-specific Parameters			
Viral load (copies/mL)	162.35 ± 79.4	-	-
CD4 count (cells/µL)	443.59 ± 90.62	-	-
HAART duration (months)	35.09 ± 13.34	-	-

Notes: Data are presented as mean ± standard deviation for continuous variables and number (percentage) for categorical variables. *p*-values were calculated using independent *t*-tests for continuous variables and chi-square tests for categorical variables. Abbreviations: PLWH, people living with HIV; HAART, highly active antiretroviral therapy; BMI, body mass index; DBP, diastolic blood pressure; SBP, systolic blood pressure. * indicates statistical significance at *p* < 0.05.

**Table 2:** Left ventricular conventional and tissue Doppler echocardiographic parameters in PLWH on HAART versus controls.

Parameters	PLWH (*n* = 118)	Controls (*n* = 80)	*p*-Values
Conventional Parameters			
LVED (mm)	47.41 ± 5.30	45.96 ± 3.90	0.198
LVES (mm)	26.63 ± 4.10	25.23 ± 3.70	0.582
IVS (mm)	10.72 ± 1.54	9.12 ± 1.50	0.135
PW (mm)	9.78 ± 1.43	9.63 ± 1.34	0.326
LVEF (%)	61.25 ± 5.32	63.08 ± 5.05	0.098
LA (mm)	34.54 ± 4.60	32.70 ± 3.40	0.068
Doppler Parameters			
E velocity (cm/s)	7.23 ± 2.87	8.68 ± 3.01	0.048 *
A velocity (cm/s)	11.33 ± 2.80	10.24 ± 3.00	0.235
E/A ratio	0.86 ± 0.42	0.77 ± 0.40	0.030 *
Tissue Doppler Parameters			
Sa velocity (cm/s)	9.40 ± 2.30	10.10 ± 2.60	0.343
IVV (cm/s)	9.30 ± 3.40	11.27 ± 3.30	0.403
AT (ms)	30.41 ± 5.10	26.97 ± 4.30	0.030 *
IVA (m/s^2^)	3.70 ± 1.60	4.30 ± 1.80	0.004 *
IVRT (ms)	76.12 ± 13.54	67.25 ± 12.00	0.098
IVCT (ms)	60.64 ± 10.40	56.32 ± 10.66	0.104
ET (ms)	214.31 ± 30.40	225.75 ± 32.10	0.027 *
MPI	0.54 ± 0.08	0.43 ± 0.10	0.001 *

Notes: Data are presented as mean ± standard deviation. *p*-values were calculated using independent *t*-tests. Abbreviations: LVED, left ventricular end-diastolic diameter; LVES, left ventricular end-systolic diameter; IVS, interventricular septum; PW, posterior wall; LVEF, left ventricular ejection fraction; LA, left atrium; E, early diastolic velocity; A, late diastolic velocity; Sa, systolic annular velocity; IVV, isovolumic velocity; AT, acceleration time; IVA, isovolumic acceleration; IVRT, isovolumic relaxation time; IVCT, isovolumic contraction time; ET, ejection time; MPI, myocardial performance index; PLWH, people living with HIV; HAART, highly active antiretroviral therapy. * indicates statistical significance at *p* < 0.05.

**Table 3:** Right ventricular function parameters and tricuspid valve Doppler measurements in PLWH on HAART.

Parameters	PLWH (*n* = 118)	Controls (*n* = 80)	*p*-Values
Conventional Parameters			
TAPSE (mm)	27.73 ± 3.50	27.11 ± 3.10	0.742
Mean PAP (mmHg)	18.51 ± 6.00	13.69 ± 5.40	0.012 *
Doppler Parameters			
E velocity (cm/s)	9.12 ± 3.20	8.58 ± 3.00	0.405
A velocity (cm/s)	12.53 ± 3.50	11.49 ± 3.10	0.340
E/A ratio	0.70 ± 0.35	0.80 ± 0.33	0.098
Tissue Doppler Parameters			
Sa velocity (cm/s)	14.91 ± 2.60	15.54 ± 2.40	0.604
IVV (cm/s)	9.68 ± 4.20	10.14 ± 4.10	0.124
AT (ms)	35.40 ± 7.00	29.35 ± 6.50	<0.001 ^†^
IVA (m/s^2^)	1.83 ± 0.95	2.49 ± 1.00	<0.001 ^†^
IVCT (ms)	69.00 ± 11.60	63.72 ± 11.00	0.054
IVRT (ms)	67.20 ± 17.30	68.00 ± 13.20	0.560
ET (ms)	174.65 ± 27.20	237.50 ± 23.50	0.006 *
MPI	0.64 ± 0.09	0.47 ± 0.08	0.010 *

Notes: Values are expressed as mean ± standard deviation. *p*-values were calculated using independent *t*-tests. Abbreviations: TAPSE, tricuspid annular plane systolic excursion; PAP, pulmonary artery pressure; E, early diastolic velocity; A, late diastolic velocity; Sa, systolic annular velocity; IVV, isovolumic velocity; AT, acceleration time; IVA, isovolumic acceleration; IVCT, isovolumic contraction time; IVRT, isovolumic relaxation time; ET, ejection time; MPI, myocardial performance index; PLWH, people living with HIV; HAART, highly active antiretroviral therapy. * indicates *p* < 0.05; ^†^ indicates *p* < 0.001.

## Data Availability

The datasets generated during this study are available from the corresponding author upon reasonable request, subject to patient privacy protections.

## References

[1] Grandominico J.M., Fichtenbaum C.J. (2008). Short-term effect of HAART on blood pressure in HIV-infected individuals. HIV Clin. Trials.

[2] Savvoulidis P., Butler J., Kalogeropoulos A. (2019). Cardiomyopathy and Heart Failure in Patients With HIV Infection. Can. J. Cardiol..

[3] Sani M.U. (2008). Myocardial disease in human immunodeficiency virus (HIV) infection: A review. Wien. Klin. Wochenschr..

[4] Walmsley S.L., Antela A.A., Clumeck N., Duiculescu D., Eberhard A.A., Gutiérrez F., Hocqueloux L.L., Maggiolo F.F., Sandkovsky U.U., Granier C.C. (2013). SINGLE Investigators. Dolutegravir plus abacavir-lamivudine for the treatment of HIV-1 infection. N. Engl. J. Med..

[5] Nan C., Shaefer M., Urbaityte R., Oyee J., Hopking J., Ragone L., Perger T., Win B., Vangerow H., McCoig C. (2018). Abacavir Use and Risk for Myocardial Infarction and Cardiovascular Events: Pooled Analysis of Data From Clinical Trials. Open Forum Infect. Dis..

[6] Depairon M., Chessex S., Sudre P., Rodondi N., Doser N., Chave J.-P., Riesen W., Nicod P., Darioli R., Telenti A. (2001). Premature atherosclerosis in HIV-infected individuals: Focus on protease inhibitor therapy. AIDS.

[7] Iacobellis G., Sharma A.M., Pellicelli A.M., Grisorio B., Barbarini G., Barbaro G. (2007). Epicardial adipose tissue is related to carotid intima-media thickness and visceral adiposity in HIV-infected patients with highly active antiretroviral therapy-associated metabolic syndrome. Curr. HIV Res..

[8] Schuster I., Thöni G.J., Edérhy S., Walther G., Nottin S., Vinet A., Boccara F., Khireddine M., Girard P.-M., Mauboussin J.-M. (2008). Subclinical cardiac abnormalities in human immunodeficiency virus-infected men receiving antiretroviral therapy. Am. J. Cardiol..

[9] Donovan C.L., Armstrong W.F., Bach D.S. (1995). Quantitative Doppler tissue imaging of the left ventricular myocardium: Validation in normal subjects. Am. Heart J..

[10] Tei C., Nishimura R.A., Seward J.B., Tajik A.J. (1997). Noninvasive Doppler-derived myocardial performance index: Correlation with simultaneous measurements of cardiac catheterization measurements. J. Am. Soc. Echocardiogr..

[11] Quiñones M.A., Otto C.M., Stoddard M., Waggoner A., Zoghbi W.A., Doppler Quantification Task Force of the Nomenclature and Standards Committee of the American Society of Echocardiography (2002). Recommendations for quantification of Doppler echocardiography: A report from the Doppler Quantification Task Force of the Nomenclature and Standards Committee of the American Society of Echocardiography. J. Am. Soc. Echocardiogr..

[12] Lang R.M., Badano L.P., Mor-Avi V., Afilalo J., Armstrong A., Ernande L., Flachskampf F.A., Foster E., Goldstein S.A., Kuznetsova T. (2015). Recommendations for cardiac chamber quantification by echocardiography in adults: An update from the American Society of Echocardiography and the European Association of Cardiovascular Imaging. J. Am. Soc. Echocardiogr..

[13] Hidayat R., Nasution S.A., Parlindungan F., Dalimunthe N.N., Alvianto S., Widjanarko N.D., Kultsum U., Efendi C., Gotama Y. (2024). Myocardial Performance Index to assess cardiac function in autoimmune connective tissue disease: A systematic review and meta-analysis. Lupus Sci. Med..

[14] Sabin C.A., Worm S.W., Weber R., Reiss P., El-Sadr W., Dabis F., De Wit S., Law M., D’Arminio Monforte A., D:A:D Study Group (2008). Use of nucleoside reverse transcriptase inhibitors and risk of myocardial infarction in HIV-infected patients enrolled in the D:A:D study: A multi-cohort collaboration. Lancet.

[15] Frerichs F.C., Dingemans K.P., Brinkman K. (2002). Cardiomyopathy with mitochondrial damage associated with nucleoside reverse-transcriptase inhibitors. N. Engl. J. Med..

[16] Louw S., Mayne E.S., Jacobson B.F., Mayne A.L. (2023). Selected inflammatory and coagulation biomarkers pre-viral suppression in people with human immunodeficiency virus (HIV) infection without overt cardiovascular disease: Is there a need to redefine reference indices?. Cytokine.

[17] Sims A., Frank L., Cross R., Clauss S., Dimock D., Purdy J., Mikhail I., Hazra R., Hadigan C., Sable C. (2012). Abnormal cardiac strain in children and young adults with HIV acquired in early life. J. Am. Soc. Echocardiogr..

[18] Reinsch N., Kahlert P., Esser S., Sundermeyer A., Neuhaus K., Brockmeyer N., Potthoff A., Erbel R., Buck T., Neumann T. (2011). Echocardiographic findings and abnormalities in HIV-infected patients: Results from a large, prospective, multicenter HIV-heart study. Am. J. Cardiovasc. Dis..

[19] Onur I., Ikitimur B., Oz F., Ekmekci A., Elitok A., Cagatay A.A., Adalet K., Bilge A.K., Kaya M.G. (2014). Evaluation of human immunodeficiency virus infection-related left ventricular systolic dysfunction by tissue Doppler strain echocardiography. Echocardiography.

[20] Sitbon O., Lascoux-Combe C., Delfraissy J.F., Yeni P., Simonneau G. (1998). Pulmonary arterial hypertension in HIV-infected patients. N. Engl. J. Med..

[21] Zola C.E., Duncan M.S., So-Armah K., Crothers K.A., Butt A.A., Gibert C.L., Kim J.W.W., Lim J.K., Re V.L., Tindle H.A. (2020). HIV- and HCV-specific markers and echocardiographic pulmonary artery systolic pressure among United States veterans. Sci. Rep..

[22] Barbaro G., Di Lorenzo G., Grisorio B., Barbarini G. (2003). Pulmonary arterial hypertension in HIV infection: A prospective echocardiographic study. AIDS..

[23] Baba M.M., Buba F., Talle M.A., Umar H., Garbati M., Abdul H. (2021). Relationship between CD4 Cell Count, Viral Load and Left Ventricular Function among HIV-1 Infected Patients Asymptomatic for Cardiac Disease on HAART. West. Afr. J. Med..

[24] Hu X., Zhang Y., Zhang T., Li W., Han J., Zhang X., Meng F. (2023). Echocardiographic assessment of left cardiac structure and function in antiretroviral therapy (ART)-naïve people living with HIV/AIDS. Immun. Inflamm. Dis..

[25] Trimarchi G., Carerj S., Di Bella G., Manganaro R., Pizzino F., Restelli D., Pelaggi G., Lofrumento F., Licordari R., Taverna G. (2024). Clinical Applications of Myocardial Work in Echocardiography: A Comprehensive Review. J. Cardiovasc. Echogr..

[26] Trimarchi G., Pizzino F., Paradossi U., Gueli I.A., Palazzini M., Gentile P., Di Spigno F., Ammirati E., Garascia A., Tedeschi A. (2024). Charting the Unseen: How Non-Invasive Imaging Could Redefine Cardiovascular Prevention. J. Cardiovasc. Dev. Dis..

